# Next-Generation Sequencing of the *Chrysanthemum nankingense* (Asteraceae) Transcriptome Permits Large-Scale Unigene Assembly and SSR Marker Discovery

**DOI:** 10.1371/journal.pone.0062293

**Published:** 2013-04-23

**Authors:** Haibin Wang, Jiafu Jiang, Sumei Chen, Xiangyu Qi, Hui Peng, Pirui Li, Aiping Song, Zhiyong Guan, Weimin Fang, Yuan Liao, Fadi Chen

**Affiliations:** College of Horticulture, Nanjing Agricultural University, Nanjing, China; Kansas State University, United States of America

## Abstract

**Background:**

Simple sequence repeats (SSRs) are ubiquitous in eukaryotic genomes. *Chrysanthemum* is one of the largest genera in the Asteraceae family. Only few *Chrysanthemum* expressed sequence tag (EST) sequences have been acquired to date, so the number of available EST-SSR markers is very low.

**Methodology/Principal Findings:**

Illumina paired-end sequencing technology produced over 53 million sequencing reads from *C. nankingense* mRNA. The subsequent *de novo* assembly yielded 70,895 unigenes, of which 45,789 (64.59%) unigenes showed similarity to the sequences in NCBI database. Out of 45,789 sequences, 107 have hits to the *Chrysanthemum* Nr protein database; 679 and 277 sequences have hits to the database of *Helianthus* and *Lactuca* species, respectively. MISA software identified a large number of putative EST-SSRs, allowing 1,788 primer pairs to be designed from the *de novo* transcriptome sequence and a further 363 from archival EST sequence. Among 100 primer pairs randomly chosen, 81 markers have amplicons and 20 are polymorphic for genotypes analysis in *Chrysanthemum*. The results showed that most (but not all) of the assays were transferable across species and that they exposed a significant amount of allelic diversity.

**Conclusions/Significance:**

SSR markers acquired by transcriptome sequencing are potentially useful for marker-assisted breeding and genetic analysis in the genus *Chrysanthemum* and its related genera.

## Introduction

SSRs (simple sequence repeats) represent an informative class of genetic markers. They are distributed throughout the coding and non-coding regions of all eukaryotic genomes [1] and have been widely use to characterize genetic diversity, identify germplasm, construct linkage maps and tag genes for the purpose of marker-assisted breeding [2]–[4]. The development of an SSR assay requires DNA sequence to design the necessary PCR primers, and sufficient such sequence is as yet lacking in most species. The development of SSRs can be a costly and time-consuming endeavor [5], [6]. One approach taken to enhance the efficiency of the process has been to target EST (expressed sequence tag) sequence, which is acquired by sequencing a reverse-transcribed preparation of mRNA. SSR markers derived from EST sequence (“EST-SSRs”) are particularly attractive because their location within coding sequence enhances the probability of successful cross-species transferability [7]–[9]. Thus, for example, when a set of EST-SSRs was generated from peach cDNA, each was shown to be functional in six other *Prunus* species [10]. Some concern has been expressed that EST-SSRs may be less informative than those targeting non-coding DNA, but in sesame at least, EST-SSRs proved to be sufficiently informative [11]. EST-SSRs therefore can simultaneously circumvent cross-species constraints and be effective in exposing polymorphism.

Next-generation sequencing (NGS) has facilitated the study of gene expression, gene regulation and gene networks in both model and non-model organisms [12]–[14]. A large volume of sequence acquired in this way has already been deposited in GenBank. In particular, NGS has enhanced the value of EST libraries by expanding sequence read lengths. As a result, EST databases have become an increasingly valuable resource for SSR marker development [6].

The Asteraceae tribe Anthemideae Cass. includes the genus *Chrysanthemum sensu lato. Chrysanthemum* species form a polyploid series (2× to 10×) based on *x* = 9. The genus is one of the largest within the Asteraceae, comprising some 1,741 species [15]. Asteraceae genomes are typically very large and highly heterozygous, so it is unlikely that any of their full genomic sequences will be acquired in the near future. To date only a small number of *Chrysanthemum* ESTs (7,180 sequences deposited in GenBank as of September 2012) has been generated, and barely any SSR markers have been developed.

The diploid species *C. nankingense* (Nakai) Tzvel is a native of China and has a relatively small genome [16], [17]. The species is particularly well adapted to environments which are exposed to either extreme temperatures (both high and low), low soil fertility and/or drought. Young leaves are consumed as a vegetable, and the plant contains cancer antagonistic flavonoids and various aromatic oils [18]. Here, we describe our effort to characterize the *C. nankingense* transcriptome, based on the use of the Illumina paired-end sequencing platform. In conjunction with the EST sequences already deposited in GenBank, the newly acquired sequences were then used to develop EST-SSR markers which should find applications from linkage mapping to marker assisted breeding in the genus *Chrysanthemum* and other related genera.

## Materials and Methods

### Plant Material and RNA Extraction

The *C. nankingense* and other germplasm used are maintained by the Chrysanthemum Germplasm Resource Preserving Centre, Nanjing Agricultural University, China. For the RNA required for the transcriptome sequencing, stems and leaves were harvested from three 30 day old *C. nankingense* cuttings rooted on MS media and grown at a constant temperature of 25°C and a 16 h photoperiod (provided by cool white fluorescent lamps producing 36 µmol m^−2^ s^−1^).

A Total RNA Isolation System (Takara, Japan) was employed to extract RNA from the plant tissue, following the manufacturer’s instructions. The quality of the RNA (RNA Integrity Number (RIN)>8.5 and 28S:18S>1.5) was verified using a 2100 Bioanalyzer RNA Nanochip (Agilent, Santa Clara, CA) and its concentration ascertained using an ND-1000 Spectrophotometer (NanoDrop, Wilmington, DE). The standards applied were 1.8≤ OD_260/280_≤2.2 and OD_260/230_≥1.8. At least 20 µg of RNA was pooled in an equimolar fashion from each of the three sample plants.

### cDNA Library Construction and Sequencing

Illumina (San Diego, CA) sequencing based on a GAII platform was performed at the Beijing Genomics Institute (Shenzhen, China; http://www.genomics.cn/index.php), following the manufacturer’s protocols. Briefly, beads coated with oligo (dT) were used to isolate poly (A) mRNA from the total RNA. A fragmentation buffer was added to interrupt the mRNA and thereby generate fragments in the size range 100–400 bp. The resulting fragments served as a template for the synthesis of the first strand cDNA, employing as primer random hexamers (N6). Second strand cDNA was synthesized using a SuperScript Double-Stranded cDNA Synthesis kit (Invitrogen, Camarillo, CA), after which it was purified using a QiaQuick PCR extraction kit (Qiagen, Hilden, Germany) and resolved with EB buffer for end reparation and poly (A) addition. The products were ligated with one another using sequencing adapters, and after agarose gel electrophoresis, a suitable size range of fragments were selected for PCR amplification. The resulting library was sequenced using an Illumina HiSeq™ 2000 device.

### Data Filtering and *de novo* Assembly

Image data output from the sequencing device were transformed into raw reads and stored in FASTQ format. These data were filtered to remove raw reads that include adapter sequence or were of low quality. The assembly of the transcriptome was achieved using the short-read assembly program Trinity [19]. The unigenes are divided into either clusters or singletons. BLASTX [20] alignment (applying an E-value of less than 10^−5^) between each unigene sequence and those lodged in Nr (non-redundant protein database, NCBI), Nt (non-redundant nucleotide database, NCBI), Swiss-Prot, GO (gene ontology, http://www.geneontology.org/) and COG (clusters of orthologous groups) databases were performed, and the best alignments used to infer the unigene’s directionality. Where the outcome from the various databases conflicted with one another, the priority order applied was Nr, Swiss-Prot, COG. Where no alignment was possible, the software tool ESTScan [21] was used to assign directionality.

### Gene Annotation and Analysis

Functional annotation was assigned using the protein (Nr and Swiss-Prot), COG and GO databases. BLASTX was employed to identify related sequences in the protein databases based on an E-value of less than 10^−5^. The COG database is an attempt to classify proteins from completely sequenced genomes on the basis of the orthology concept [22]. GO’s aim is to standardize the representation of genes and their products, by insisting on a controlled vocabulary and a strictly defined concept [23], [24]. The annotations acquired from Nr were processed through the Blast2GO program [25] to obtain the relevant GO terms, and these were then analyzed by WEGO software [26] to assign a GO functional classification and to illustrate the distribution of gene functions.

### Development of cDNA-derived SSR Markers and Primer Design

Unigenes containing putative SSRs were identified by MISA software (http://pgrc.ipk-gatersleben.de/misa/), applying the following parameters: a minimum of six repeats for dinucleotide motifs, of five for trinucleotides, of four for tetranucleotides and of three for penta- and hexa-nucleotides. Primer 3.0 software (http://sourceforge.net/projects/primer3) was used to design appropriate primers from the flanking sequence, based on the following criteria: primer length 18–22 bp (optimally 20 bp), T_m_ of 50–60°C (no more than a 4°C difference between the T_m_s of the forward and reverse primers) and an amplicon length in the range 100–400 bp. Primers were synthesized by Invitrogen (Shanghai, China).

### EST-SSR Screening and Polymorphism Survey

Genomic DNA was isolated from leaves following published protocols [27]. The DNA was dissolved in 50 µl water, diluted to a final concentration of 10 ng/µl and stored at −20°C until required. Genetic diversity was analyzed by tailing the forward SSR primer with a fluorescently labeled M13 sequence (CACGACGTTGTAAAACGAC) [28]. Agarose gel electrophoresis separation of the amplicons produced at 100 randomly chosen SSR loci from *C. nankingense* DNA template was used to check the functionality of the primers. A set of 20 of the 81 functional primer pairs was then used to amplify the DNA of 42 commercial chrysanthemum varieties and 30 related wild species (Table 1). The PCR conditions comprised an initial denaturing step (95°C/3 min), followed by ten cycles of 94°C/30 s, 50–60°C/30 s, 72°C/30 s, then 25 cycles of 94°C/30 s, 55°C/30 s, 72°C/30 s, and finally by an elongation step (72°C/7 min). The amplicons were separated using an ABI3730xl (Applied Biosystems) device, following the manufacturer’s protocols. Each sample was supplemented with ABI GeneScan LIZ500 size standard in order to determine amplicon lengths with the aid of GeneMapper® v3.7 software (Applied Biosystems). For the statistical analysis, the patterns at all SSR loci were scored for each polymorphic band as 1 for band presence and 0 for band absence. This allowed an estimate at each locus of the number of alleles present (NA) and the PIC value. Similarity coefficients based on SSR profiles were calculated according to Nei and Li [29]. Genetic relationships between materials were examined using cluster analysis implemented in the NTSYS-pc2.1 software package and a dendrogram was constructed based on the unweighted pair group method of arithmetic average algorithm. Some representative PCR products were also separated through 6% denaturing polyacrylamide gels (19∶1 acrylamide: bisacrylamide, 7.5 M urea, 1 × Tris-borate-EDTA pH 7.8) and visualized by silver staining.

**Table 1:** SSR genotypes of species within the genera Chrysanthemum, Leucanthemella, Ajania, Artemisia, Opisthopappus, Pyrethrum, Tanacetum and Crossostephium.

Plant lines	Primer pairs																			
	70	86	88	89	99	123	135	170	179	204	214	245	249	254	271	285	312	313	320	341
***Chrysanthemum dichrum***	1	1	1	1	1	1	1	1	1	1	1	1	1	1	1	1	1	1	1	1
***Chrysanthemum lavandulifolium***	1	1	1	1	1	1	1	1	1	1	1	1	1	1	1	○	1	1	1	1
***Chrysanthemum boreale***	1	1	1	1	1	1	1	1	1	1	1	1	1	1	1	1	1	1	1	1
***Chrysanthemum japonicum***	1	N	1	1	1	1	1	1	1	1	1	1	1	N	1	1	1	1	1	1
***Chrysanthemum zawadskii***	1	1	1	1	1	1	1	1	1	1	1	1	1	1	1	1	1	1	1	1
***Chrysanthemum chanetii***	1	1	1	1	1	1	1	1	1	1	1	1	1	1	1	○	1	1	1	1
***Chrysanthemum indicum***	1	N	1	N	1	1	1	1	N	○	1	1	1	1	1	1	1	1	1	1
***Chrysanthemum yoshinaganthum***	1	1	1	1	1	1	1	1	1	1	1	1	1	1	1	1	1	1	1	1
***Chrysanthemum ornatum***	1	1	1	N	1	1	1	1	1	1	1	○	1	1	1	○	1	1	1	1
***Chrysanthemum vestitum***	1	1	1	N	1	1	1	1	1	1	1	1	1	1	1	○	1	1	1	1
***Chrysanthemum morifolium***	1	1	1	1	1	1	1	1	1	1	1	1	1	1	1	1	1	1	1	1
***Chrysanthemum japonense***	1	1	1	N	1	○	1	1	1	1	1	1	1	1	1	1	1	1	1	1
***Chrysanthemum crassum***	1	1	1	1	1	1	1	1	1	1	1	1	1	1	1	1	1	1	1	1
***Ajania przewalskii***	1	1	1	1	1	1	1	1	1	1	1	○	1	1	1	1	1	1	1	1
***Ajania myriantha***	1	1	1	1	1	1	1	1	1	1	1	1	1	1	1	1	1	1	1	1
***Ajania.pacificum***	1	1	1	1	1	1	1	1	1	N	1	1	1	1	1	1	1	1	1	1
***Ajania.shiwogiku***	1	1	1	1	N	1	1	1	1	1	1	1	1	1	1	1	1	1	1	1
***Ajania shiwogiku***	1	1	1	1	1	1	1	1	1	1	1	1	1	1	1	1	1	1	1	1
***Artemisia sieversiana***	1	1	1	1	1	1	1	1	1	1	1	1	1	1	1	1	1	1	1	1
***Artemisia annua***	1	1	1	1	1	1	1	1	1	1	1	1	1	N	1	1	1	1	1	1
***Artemisia absinthium***	1	1	1	1	1	1	1	N	N	1	1	1	1	N	1	1	1	1	1	1
***Artemisia vulgaris*** ** (L.)**	1	1	1	1	1	1	1	1	1	1	1	1	1	N	1	1	1	1	1	1
***Artemisia japonica***	1	1	1	○	1	1	1	1	1	1	1	1	1	N	1	1	1	1	1	1
***Artemisia sericea***	1	1	1	○	1	1	1	1	1	1	1	1	1	N	1	1	1	1	1	1
***Opisthopappus Taihangensis***	1	1	1	1	1	1	1	1	1	1	1	1	1	1	1	1	1	1	1	1
***Crossostephium chinense***	1	1	1	1	1	1	1	1	1	1	1	1	1	1	1	1	1	1	1	1
***Tanacetum vulgare***	1	1	1	1	1	1	1	1	1	1	1	1	1	N	1	1	1	1	1	1
***Pyrethrum parthenium***	1	1	1	1	1	1	1	1	1	1	1	1	1	1	1	1	1	1	1	1
***Leucanthemella linearis***	1	1	1	1	1	1	1	1	1	1	1	1	1	1	1	1	1	1	1	1

Note: 1: Amplicon different from that of *C. nankingense,* N: No amplification, O: Amplicon indistinguisable from that of *C. nankingense.*

## Results

### Sequencing and *de novo* Assembly

**Figure 1:**
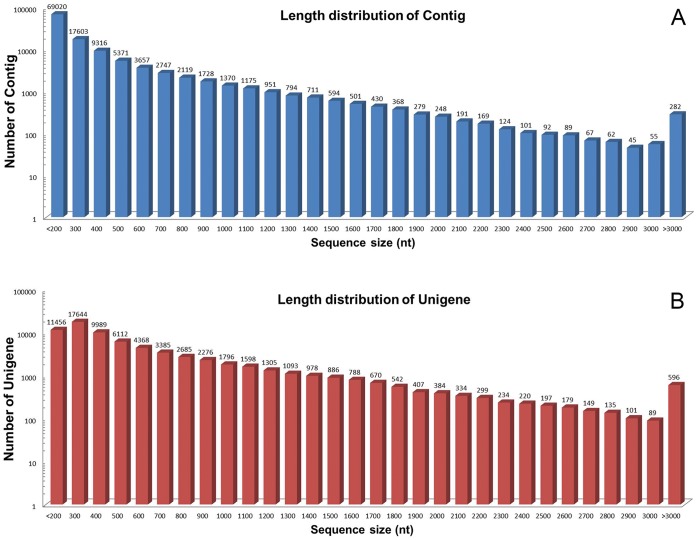
The distribution of contig and unigene sequence lengths.

In all, 53,720,166 sequence reads were generated, of which 51,622,828 were of acceptable quality (Table 2). The *de novo* assembly yielded 120,260 contigs of mean length 321 bp (Fig. 1A), and these were resolved into 70,895 unigenes, of which 26,650 were clusters and 44,245 singletons (of length at least 150 bp). The range in unigene length was from 150 bp to 9,032 bp (mean 585 bp) (Fig. 1B).

**Table 2:** Summary of sequencing output statistics.

Samples	Total Raw Reads	Total Clean Reads	Total Clean Nucleotides	Q20%	N %	GC %
*C. nankingense*	53,720,166	51,622,828	4,646,054,520	98.37%	0.00%	45.05%

* Total Clean Nucleotides = Total Clean Reads1×Read1 size+Total Clean Reads2×Read2 size.

### Structural and Functional Annotation

**Figure 2:**
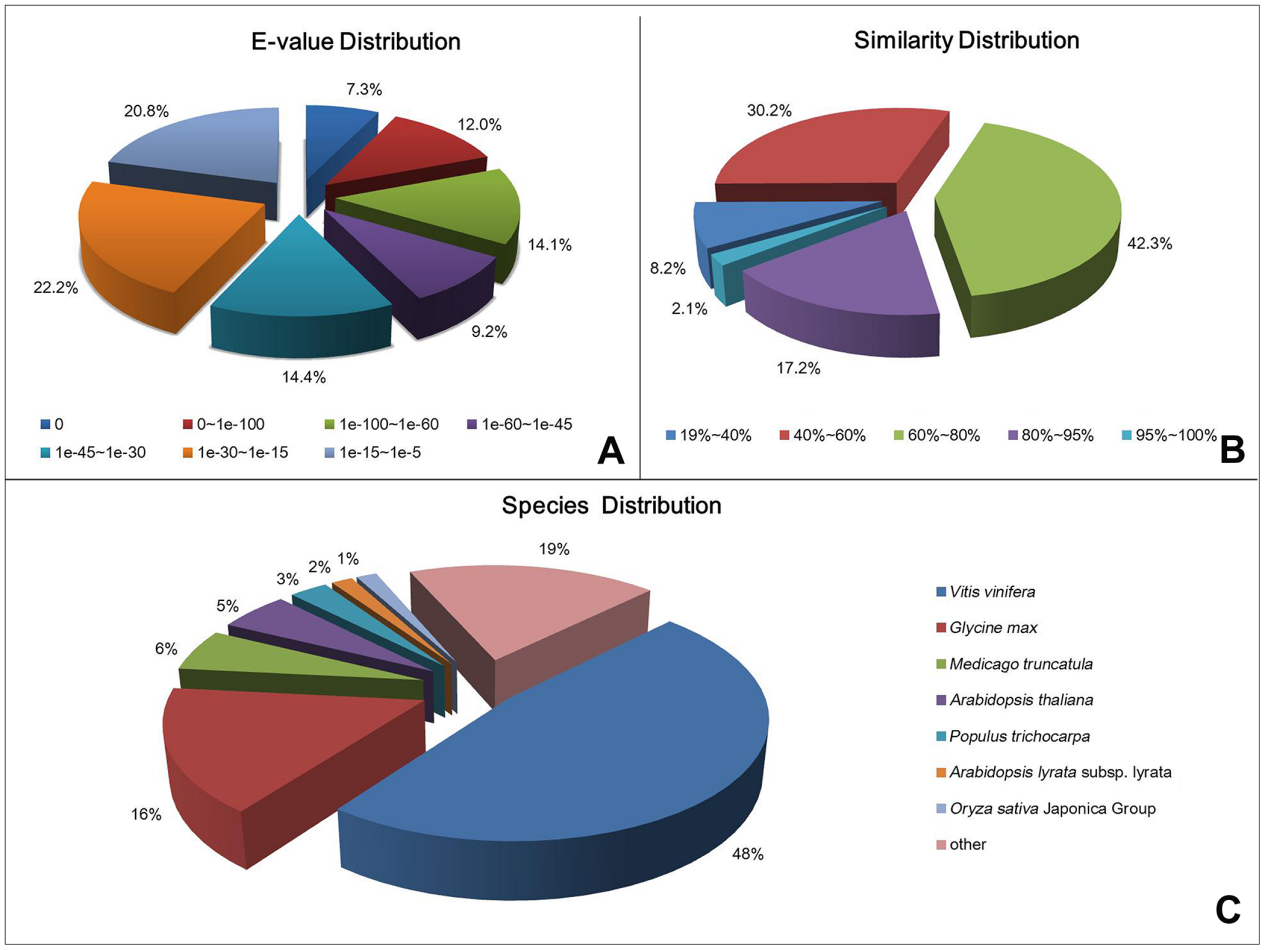
The distribution of E-value, sequence similarity, and species of data base hits with the *C. nankingense* unigenes.

A total of 45,789 of the unigene sequences shared some similarity to known genes. The three most frequently encountered transcripts encoded a light-regulated protein, a catalase and an arabinogalactan peptide. Just 107 of the unigenes showed sequence homology with the *Chrysanthemum* spp. sequences lodged in the Nr database, and the number of hits with sunflower and lettuce (also Asteraceae species) sequences was, respectively, 679 and 277. The ranges in E-value and sequence similarity of the top hits in the Nr database were comparable, with 33.4% (E-value of 0 to −60) and 19.3% (100%–80%) of the sequences possessing homology (Fig. 2A, B). On a species basis, the highest proportion of matching sequences in the Nr database were derived from grapevine (47.7%), followed by soybean (16.5%) (Fig. 2C).

**Figure 3:**
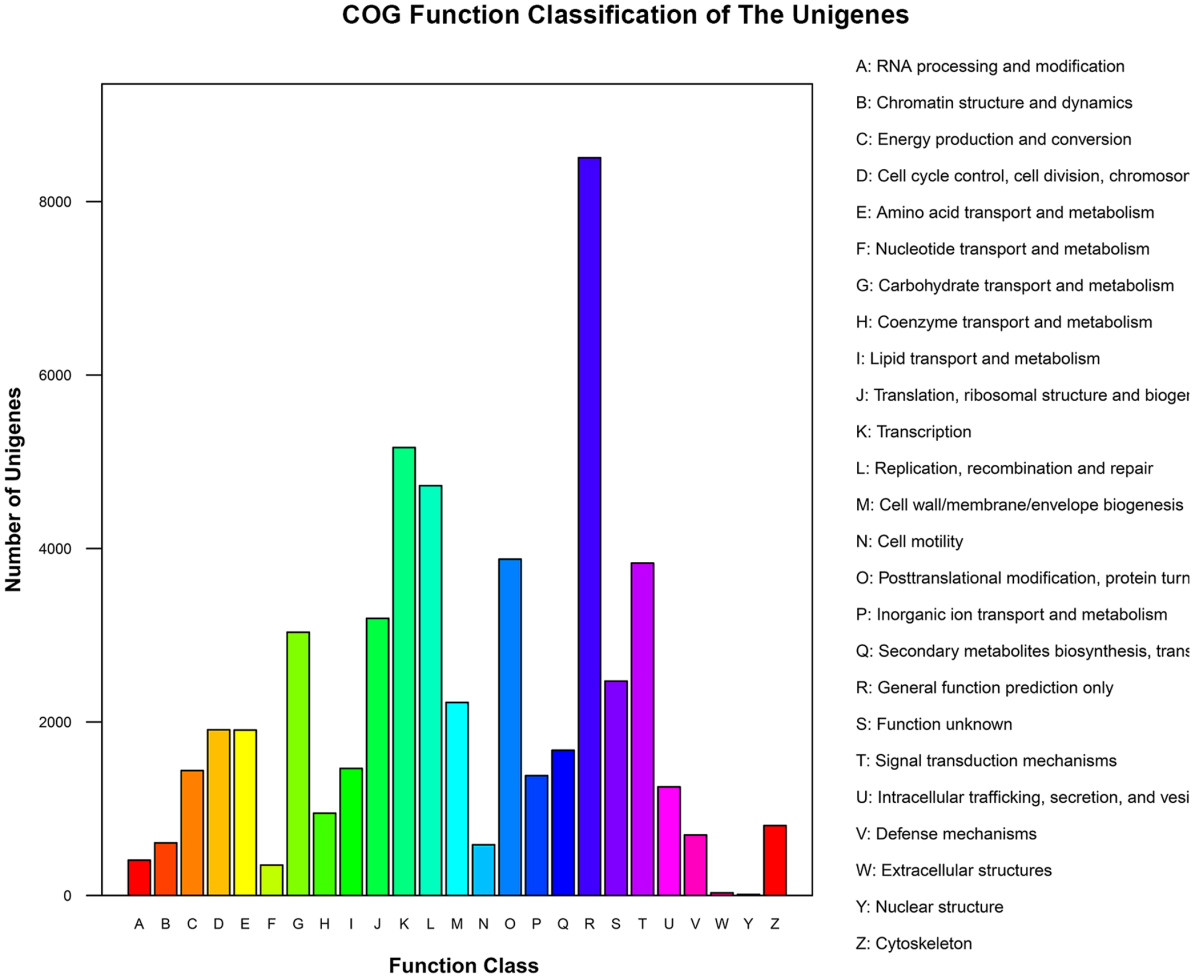
Functional classification of the *C. nankingense* unigenes according to COG criteria.

**Figure 4:**
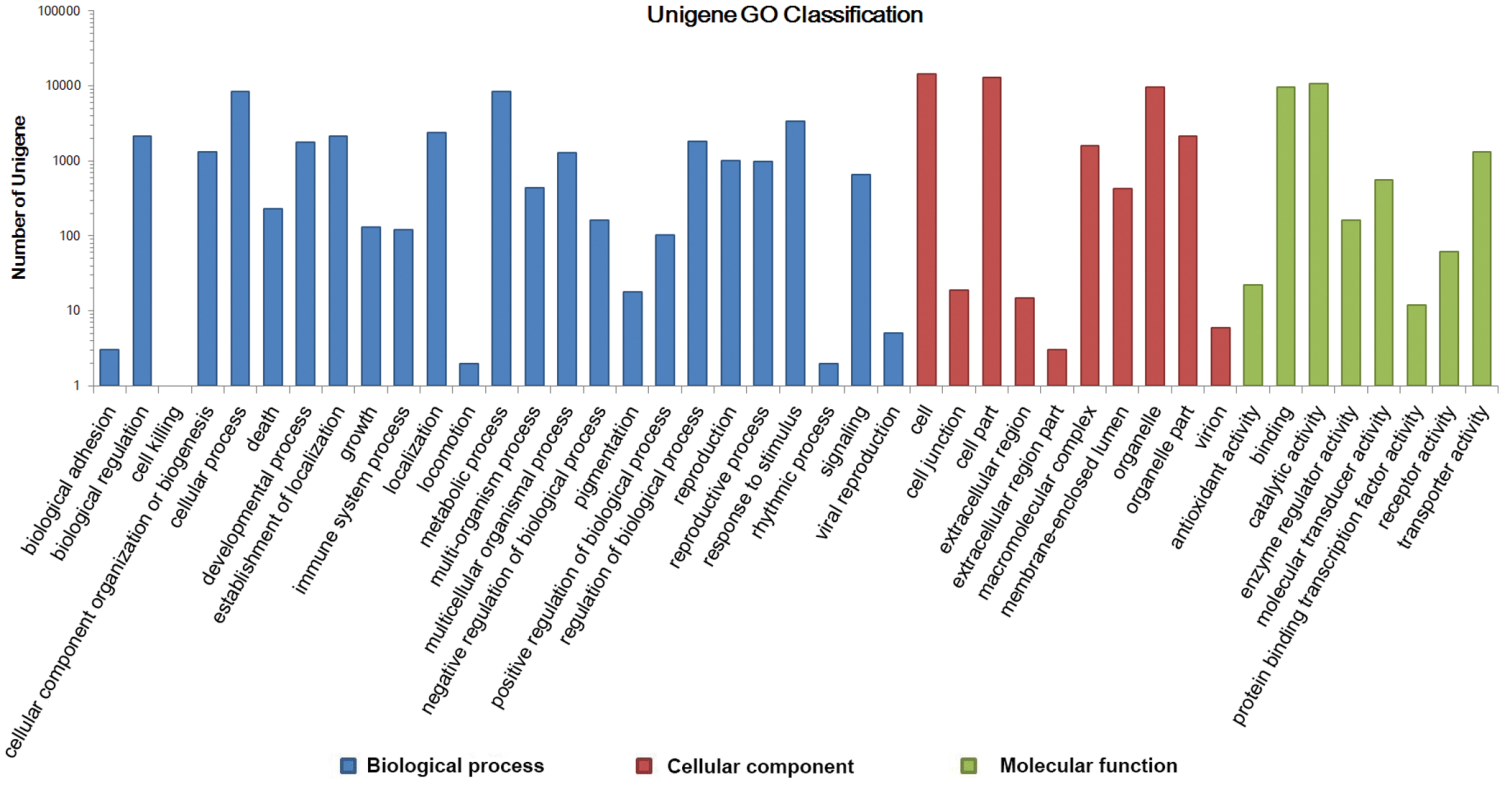
The distribution of *C. nankingense* unigenes among the GO functional classes. From left to right, “Biological process” (shown in *blue*): biological adhesion, biological regulation, cell killing, cellular component organization or biogenesis, cellular process, death, developmental process, establishment of localization, growth, immune system process, localization, locomotion, metabolic process, multi-organism process, multicellular organismal process, negative regulation of biological process, pigmentation, positive regulation of biological process, regulation of biological process, reproduction, reproductive process, response to stimulus, rhythmic process, signaling, viral reproduction; “Cellular components” (shown in *red*): cell, cell junction, cell part, extracellular region, extracellular region part, macromolecular complex, membrane-enclosed lumen, organelle, organelle part, virion; “Molecular function” (shown in *green*): antioxidant activity, binding, catalytic activity, enzyme regulator activity, molecular transducer activity, protein binding transcription factor activity, receptor activity, transporter activity.

The COG analysis allowed the functional classification of 21,952 of the unigenes (Fig. 3). The most frequently identified classes were “general function” (4,503, 16.3%), followed by “transcription” (2,622, 9.5%), “replication, recombination and repair” (2,370, 8.6%), “post-translational modification, protein turnover and chaperones” (2,189, 7.9%), “signal transduction” (1,898, 6.9%) and “translation, ribosomal structure and biogenesis” (1,714, 6.2%). Nr annotation assigned 36,985 of the unigenes to the category of “biological process”; within this ontology, the two most common functions were “cellular process” (8,639, 22.6%) and “metabolic process” (8,502, 23.0%). At the level of localization, “cell” applied to 14,402 unigenes (34.8%), “cell part” to 13,003 (31.4%) and “organelle” to 9,765 (23.6%). Although the function of the unigenes covered a comprehensive range of GO categories, “catalytic activity” (10,724, 47.5%) and “binding” (9,738, 43.1%) proteins made up the majority, while “transporter activity”, “molecular transducer activity”, “enzyme regulator activity”, “receptor activity”, “antioxidant activity” and “protein binding transcription factor activity” proteins together made up only 9.4% (Fig. 4).

### Development and Characterization of cDNA-derived SSR Markers

**Figure 5:**
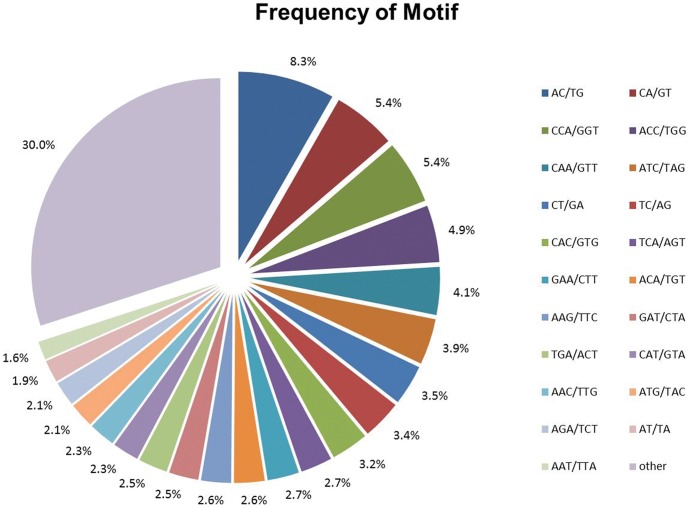
Frequencies of the various repeat motifs present in the *C. nankingense* EST-SSRs.

MISA analysis identified 2,813 putative microsatellites, equivalent to one locus per 14.7 kb of the *C. nankingense* transcriptome. Based on the length of the repeat motif, the sequences were into two groups: Class I were hypervariable markers, consisted of SSRs ≥20 bp; Class II, or potentially variable markers were consisted of SSRs 12–20 bp [30], [31]. In all, 1,788 primer pairs were designed (Table S1), of which 342 (19.1%) targeted Class I loci and the remaining ones Class II loci. Almost all the sequences (95.6%) shared high homology to known genes. A further 501 putative SSRs were located among the EST sequences lodged in GenBank; from these, 363 PCR primer pairs were designed (Table S1). The most abundant repeat motifs were trinucleotides (1,914, 57.8%), followed by dinucleotides (747, 22.5%) and tetranucleotides (243, 7.3%). Over 160 motifs were identified, of which the most frequent were AC/TG (8.3%), CA/GT (181, 5.4%), CCA/GGT (181, 5.4%), ACC/TGG (162, 4.9%), CAA/GTT (131, 4.1%) and ATC/TAG (3.9%) (Fig. 5).

### Polymorphism Survey with EST-SSR

**Figure 6:**
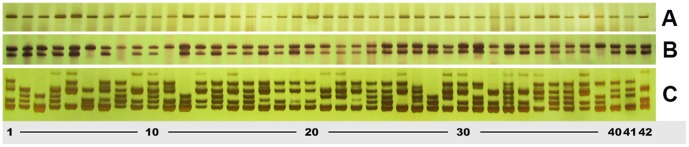
SSR allelic variation among 42 commercial *C. morifolium* cultivars. (A) Single product, non-polymorphic amplicon(primer #214), (B) Two product, polymorphic amplicon (primer #86), (C) Multiple product, highly polymorphic amplicon (primer #245). Lanes 1–42: cv. ‘Zhongshanzigui’, cv. ‘Zhongshanjingui’, cv. ‘Zhongshanhongying’, cv. ‘Zhongshanaihuang’, cv. ‘Zhongshanzihe’, cv. ‘Zhongshanchengguang’, cv. ‘Zhongshanhongfeng’, cv. ‘Zhongshanhuangtuogui’, cv. ‘Zhongshanguohuang’, cv. ‘Zhongshanbailu’, cv. ‘Zhongshanfenzhuang’, cv. ‘Zhongshanzilian’, cv. Zhongshanfendai’, cv. ‘Zhongshanqueyu’, cv. ‘Zhongshanhongxia’, cv. ‘Zhongshanhuangying’, cv. ‘Zhongshanhuangyu’, cv. ‘Zhongshanjinyu’, cv. ‘Zhongshanyinxing’, cv. ‘Zhongshanzaobai’, cv. ‘Zhongshanzixing’, cv. ‘Zhongshanziyu’, cv. ‘Zhongshanfengui’, cv. ‘Zhongshanjinzhen’, cv. ‘Zhongshanxuegui’, cv. ‘Zhongshanzuirong’, cv. ‘Jinlingaihuang’, cv. ‘Jinlingbaifeng’, cv. ‘Jinlingbaixue’, cv. ‘Jinlingbaiyu’, cv. ‘Jinlingbaohui’, cv. ‘Jinlingbaoxia’, cv. ‘Jinlingchixin’, cv. ‘Jinlingchunse’, cv. ‘Jinlingfanxing’, cv. ‘Jinlingfendai’, cv. ‘Jinlingguofen’, cv. ‘Jinlingguohong’, cv. ‘Jinlingguohuang’, cv. ‘Jinlingguozi’, cv. ‘Jinlinghonghe’, cv. ‘Jinlinghongpao’.

**Figure 7:**
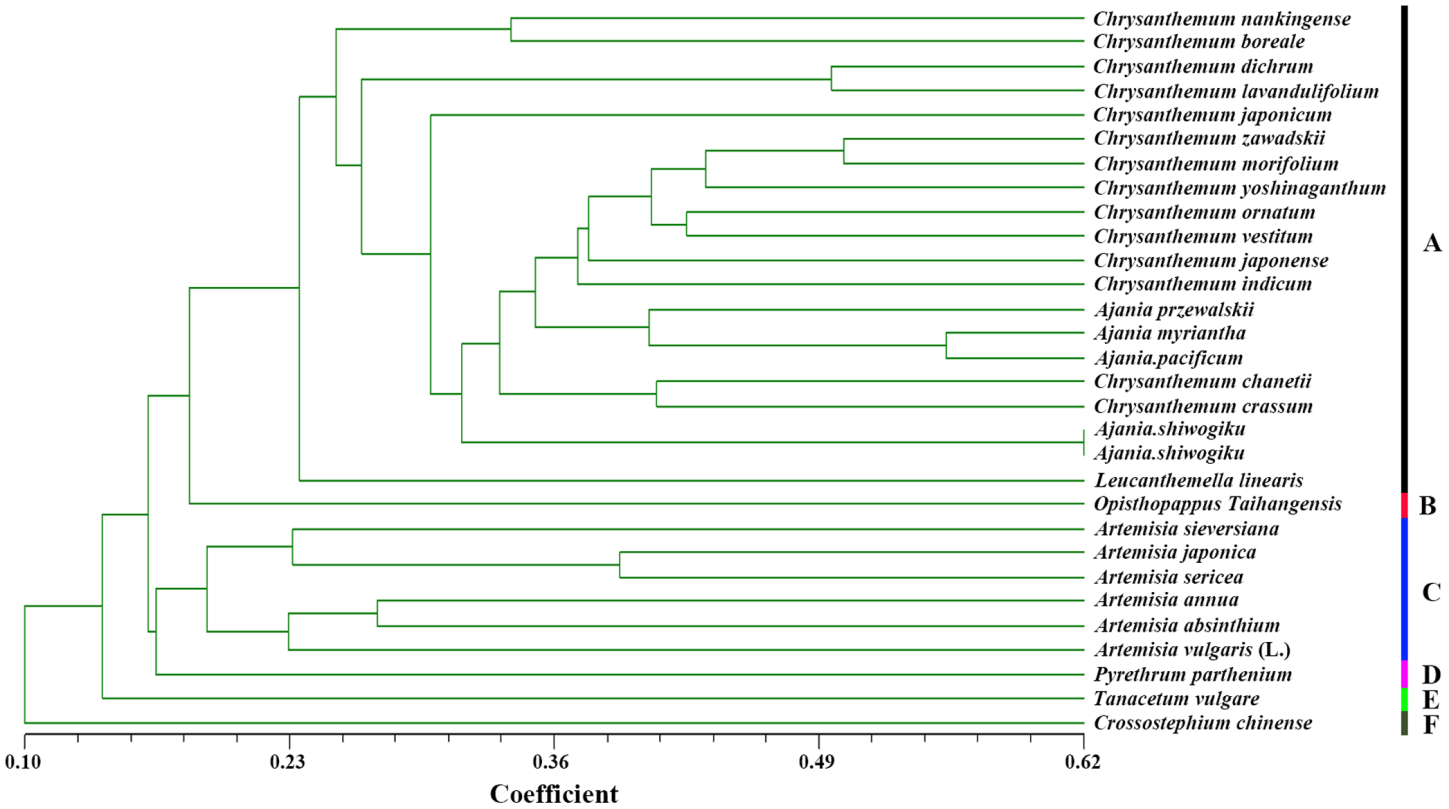
UPGMA-based phylogeny of *Chrysanthemum* spp. and species belonging to closely related genera. The tree was derived from genotype data from 20 EST-SSR loci. (A–F) Six clades were recognized.

All 20 primer pairs (targeting 16 Class I and four Class II loci) have amplicons in 42 chrysanthemum cultivated varieties and could be used for marker-assisted breeding in chrysanthemum (Fig. 6). However, not all of them amplified the DNA of all 30 wild relatives. For example, even though most wild materials displayed the polymorphism, we could not detect any amplicon bands using primer pair 170 in *Artemisia absinthium*. Nearly all of the templates - but not those of several of the *Artemisia* spp. - amplified a product using primer pair #254 (Table 1). The phylogeny of the wild species based on their EST-SSR genotype using 20 of the primer pairs (Fig. 7) illustrates that they fall into six clades. The set of *Chrysanthemum* and *Ajania* spp., along with *Leucanthemella linearis*, formed one clade, the *Artemisia* spp. a second clade, leaving the *Opisthopappus*, *Pyrethrum*, *Tanacetum* and *Crossostephium* spp. separated both from one another and from each of the two major clades.

## Discussion

### Sequencing, Assembly and Annotation

NGS has been widely used for *de novo* transcriptome sequencing, especially in non-model organisms [32]–[37]. With respect to Asteraceae species, the representation of ESTs comprises sequences from lettuce, sunflower and gerbera. The current *C. nankingense* data consisting of 70,895 unigene sequences is comparable in size to the 81,330 lettuce EST collection, and larger than that of either *Centaurea solstitialis* (40,407) or *Zinnia violacea* (20,767) (http://www.ncbi.nlm.nih.gov/dbEST/dbEST_summary.html). It is also noteworthy that 44,245 of the *C. nankingense* unigenes matched genes with unique annotations, a proportion which is comparable with what has been experienced in diploid plant species, e. g., rice, *Arabidopsis thaliana* and *Brachypodium distachyon* [38], [39]. Unigene sequences have a number of applications, such as the identification of single nucleotide polymorphism markers [40], [41], alternative gene splicing products [42,42], homologous genes [43]–[45] and gene families [46], [47]. Over 64% of the products of the unigenes shared homology with known proteins, a proportion which is rather higher than in gerbera, where only 42% of unigene sequences satisfied this criterion [48]. About 80.8% of the annotated unigenes could be assigned to the category “biological process”, which indicates that a wide diversity of transcripts is represented in the data set. Thus this sequence resource should have utility in future efforts to either identify novel genes or to undertake genome-wide transcriptomic analyses in chrysanthemum.

### Identification and Characterization of SSR Marker Sequences

The development of informative SSRs remains a labor intensive process [49], [50]. Mining the gerbera sequence for functional SSR markers only realised 99 assays [51], although 730 potentially informative assays were generated for the Asteraceae species *Cichorium intybus* [52]. Here we report the development of 1,788 and 363 EST-SSRs based on present transcriptome and previously published ESTs in GenBank, respectively. To our knowledge, this represents the first reported mass isolation of EST-SSRs in *Chrysanthemum.* NGS clearly offers a rapid means of acquiring the sequence needed to discover SSRs and to design the necessary primers to form an assay. EST-SSR markers are of particular interest, as allelic variants can entail alterations in the coding sequence which could underlie specific phenotypic variation [53], [54]. EST-SSR markers also tend to have a higher amplification efficiency and are more likely to be transferable across species than is the case for SSR markers derived from non-coding regions of the genome [55]. The frequency of EST-SSR loci and their ease of PCR amplification were consistent with the experience of other plant species [4], [24], and it was relatively easy to generate a substantial number of informative markers of use both within cultivated chrysanthemum germplasm and among its wild relatives. The overall results showed that nearly all plant materials have genetic polymorphism, suggesting that the regions flanking the repeats are highly conserved.

Several lines of evidence suggest that the genera *Leucanthemella*, *Ajania* and *Chrysanthemum* are closely related to one another, but that *Artemisia* is rather distant [56], [57]. The phylogenetic relationships between these genera inferred from ETS-SSR genotype were largely consistent with this picture. *Opisthopappus* is a recently described Compositae genus from China; it differs from *Tanacetum* in a number of morphological ways [58]. Meanwhile, *Crossostephium* has been defined as a small separate genus, distinct from *Pyrethrum* [59]. The EST-SSR phylogeny confirmed that *Opisthopappus*, *Pyrethrum*, *Tanacetum* and *Crossostephium* are independent genera, and was congruent with phylogenies based on variation in the nuclear ribosomal ITS and chloroplast *trn*L-F IGS sequences [60]. All those data validated the universality of the 20 randomly selected EST-SSRs.

### Conclusions

The Asteraceae species form a large and poorly understood group of plants, which nevertheless contains a number of horticulturally significant species. Nearly all the SSR assays developed were able to distinguish the cultivated *C. morifolium* germplasm from the wild relative species. The markers described here should find application in a range of research activity, including the assessment of genetic diversity, the identification of germplasm, the construction of linkage maps and gene tagging for marker-assisted breeding. Any genomic variable that affects genetic function may have an evolutionary role more or less. Some experimental evidence has suggested that the rapid evolution of SSR sequences - specifically, the gain or loss of repeats at a locus - may provide a molecular basis for adaptation to unfamiliar environments [61], [62]. Whether or not this holds for the Asteraceae will require further research.

## Supporting Information

Table S1 **Microsatellite markers developed from the** ***de novo*** **transcriptome sequence of** ***C. nankingense*** **.**
(XLS)

Table S2 **Microsatellite markers developed from the NCBI dbEST database.**
(XLS)
